# A polysaccharide isolated from *Agaricus blazei* Murill (ABP-AW1) as a potential Th1 immunity-stimulating adjuvant

**DOI:** 10.3892/ol.2013.1484

**Published:** 2013-07-23

**Authors:** LIRAN CUI, YONGXU SUN, HAO XU, HUIYU XU, HUAN CONG, JICHENG LIU

**Affiliations:** 1 Department of Medicine Research, The First Affliated Hospital, Qiqihar Medical University, Qiqihar, Heilongjiang 161041, P.R. China; 2 Heilongjiang University of Chinese Medicine, Harbin, Heilongjiang 150040, P.R. China; 3 Department of Medicinal Chemistry and Biomacromolecules, Qiqihar Medical University, Qiqihar, Heilongjiang 161006, P.R. China

**Keywords:** polysaccharide, *Agaricus blazei*, Th1, vaccine, immune

## Abstract

In the present study, a low molecular weight polysaccharide, ABP-AW1, isolated from *Agaricus blazei* Murill was assessed for its potential adjuvant activity. ABP-AW1 is considered to create a ‘depot’ of antigen at a subcutaneous injection site. ICR mice were immunized with 100 μg ovalbumin (OVA) alone or with 100 μg OVA formulated in 0.9% saline containing 200 μg aluminum (alum) or ABP-AW1 (50, 100 and 200 μg) on days 1 and 15. Two weeks after the secondary immunization, splenocyte proliferation, the expression of surface markers, cytokine production and the OVA-specific antibody levels in the serum were determined. The OVA/ABP-AW1 vaccine, in comparison with OVA alone, markedly increased the proliferation of splenic lymphocytes and elicited greater antigen-specific CD4^+^ T cell activation, as determined by splenic CD4^+^CD69^+^ T cells and Th1 cytokine interferon (IFN)-γ release. The combination of ABP-AW1 and OVA also enhanced IgG2b antibody responses to OVA. In conclusion, these data indicated that ABP-AW1 significantly enhanced the humoral and cellular immune responses against OVA in the mice, suggesting that ABP-AW1 stimulated Th1-type immunity. We suggest that ABP-AW1 may serve as a new adjuvant.

## Introduction

Vaccines greatly reduce the spread and mortality of infectious diseases and cancer. The ability of vaccines to counteract infections and fight against cancer mainly involves a three-tiered functionality; humoral and cellular immunity and regulators of the immune system, such as cytokines (1). Vaccines usually require additional exogenous adjuvants to improve the immune response to the antigens following immunization (2). Hence, one of the most significant challenges in vaccinology is the selection of suitable adjuvants (3). The aluminum (alum) adjuvant has been successfully used in millions of humans as the principle adjuvant in clinical vaccines since 1932 and greatly decreases morbidity and mortality with minimal toxicity (2). However, alum is a relatively poor adjuvant in numerous situations, particularly when used with subunit antigens (4). Furthermore, alum is not highly effective at stimulating cell-mediated immune responses (5), including the vaccination against pathogens that require Th1-cell-mediated immunity (2). Freund’s complete adjuvant (6,7) promotes a marked commitment to the Th1 pathway, but has a generally unacceptable level of adverse effects. The MF59 oil-in-water emulsion adjuvant is the only other adjuvant approved for human use besides alum, although it has been reported to favor only the Th2 immune response, similar to alum (8). In addition, alum is not valid for the induction of mucosal IgA antibody responses and certain studies have shown that alum is associated with allergic reactions in certain subjects (3). There is no controversy over the fact that solving these issues should aid in improving the effectiveness of alum salts and in the development of alternative adjuvants, as there is an urgent requirement for adjuvants capable of boosting Th1-type responses without unacceptable toxicity.

Carbohydrate structures are renowned for their ability to boost immune function and have the virtue of maximum tolerability and safety (9). Moreover, they are readily biodegradable, with a low risk of generating toxic metabolites (10,11). A large number of carbohydrate compounds have emerged as promising vaccine adjuvant candidates. Among the great range of carbohydrate structures, polysaccharides stand out as the most promising as they are less likely to cause adjuvant accumulation and excessive and chronic immune activation (9). Polysaccharide-rich fungi and plants have attracted growing scientific interest for their dietary and medicinal benefits (12–14), as well as for their ability to exert marked effects on immune system function, inflammation and cancer (12,13,15), Such polysaccharides have also been used as intranasal adjuvants for the induction of mucosal and systemic immune responses (16,17). This provides a foundation that may aid in guiding future research on immune modulation by well-characterized polysaccharide compounds. However, the development of vaccines based on polysaccharides must overcome certain drawbacks and improve the effectiveness of polysaccharides. For example, children below two years old and the elderly respond poorly to polysaccharide antigens due to the immaturity or aging of the immune system (18). Hence, there is an urgent requirement for the use of polysaccharide adjuvants, in particular the rational development of new adjuvants and immunostimulators for vaccines.

*Agaricus blazei* Murill is a traditional Chinese fungus that possesses numerous pharmacological properties (19), including the modulation of biological homeostasis in order to boost the ability to fight infection, counteract diseases and prevent cancer (20–22). Previously, we reported that a polysaccharide from *Agaricus blazei* was able to suppress tumor growth and angiogenesis *in vivo*(23), inhibit sialyl Lewis X/E-selectin-mediated metastasis in HT-29 cells (24) and prevent and attenuate hematogenous metastasis in HT-29 cells (25). However, to date, no information has been published concerning *Agaricus blazei* polysaccharides as potential adjuvants. We previously identified a new intracellular polysaccharide, ABP-AW1 (Mw, 50 kDa), from *Agaricus blazei* Murill (26). The structural features of the purified polysaccharide were successfully characterized by means of chemical analyses and instrumental spectroscopy. These results showed that the backbone chains of ABP-AW1 are mainly (1→6)-linked-β-D-galactopyranose, (1→6)-linked-β-D-glucopyranose and (1→3,6)-linked-β-D-glucopyranose, terminating with (1→)-linked Fuc, Ara and Man residues at the O-3 position of (1→3,6)-linked-β-D-glucopyranose in the proportion of 29:10:10:6:2:2. Hence, in the present study, we hypothesized that ABP-AW1 is able to act as an adjuvant for the development of adaptive immunity. To test this hypothesis, the *in vivo* adjuvant activity of the ABP-AW1 adjuvant system was evaluated using ovalbumin (OVA) as a model protein antigen. The aim was to determine whether ABP-AW1 was able to enhance the cellular immunity, in addition to the humoral immunity, of mice subcutaneously immunized with OVA, particularly the Th1-type responses.

## Materials and methods

### Mice

Male ICR mice (Grade II, five to six weeks old) were obtained from Jilin University Animal Research Center (Changchun, China). The mice were specific pathogen-free and acclimated for one week prior to use. Rodent laboratory chow and tap water were provided *ad libitum* and the mice were exposed to a 12 h/12 h light/dark cycle at 24±1°C and 50±10% relative humidity. All animal procedures were performed according to the Guide for the Care and Use of Laboratory Animals of the National Research Council. This study was approved by the Animal Care and Use Committee of Qiqihar Medical University (Qiqihar, China).

### Isolation and purification of polysaccharide

The ABP-AW1 polysaccharide was prepared and characterized as previously described (26). Briefly, *Agaricus blazei* Murill were subjected to three treatments with 95% ethanol (5,000 ml) at 75°C for 6 h under reflux to remove any lipids. The residue was extracted three times with distilled water (8,000 ml) at 75°C for 3 h. The supernatant was removed and the precipitate was washed, dried and extracted twice with 0.5 M NaOH solution, containing 0.3% (w/w) NaBH_4_ at room temperature, which was incubated overnight. Subsequent to filtering to remove the debris, the suspension was neutralized with hydrochloric acid (0.1 M) and filtered. The supernatant containing water-soluble polysaccharide was dialyzed, concentrated, ethanol precipitated and dried. The precipitate was obtained by centrifugation (4,000 × g for 10 min) and deproteinized by the Sevag method (27), followed by exhaustive dialysis with distilled water for 48 h. The concentrated dialyzate was then precipitated at 4°C with 95% ethanol (4,000 ml) for 24 h. The precipitate was washed with absolute ethanol, acetone and ether, yielding the aqueous crude polysaccharide (CABP-AW).

The CABP-AW was redissolved with distilled water and centrifuged at 4,000 × g for 10 min, then the supernatant was applied to a DEAE Sepharose Fast Flow column (Amersham Biosciences, Uppsala, Sweden) that had been equilibrated with ultrapure water. After loading the sample, the column was eluted stepwise with NaCl aqueous solution (0, 0.2, 0.4 and 0.6 M) at a flow rate of 4 ml/min. The fractions (8 ml) were collected using a Frac-950 (Amersham Biosciences) and the polysaccharide was purified further by gel-permeation chromatography on a Sepharose 6 Fast Flow column (2.6×100 cm) with 0.15 M NaCl at a flow rate of 1 ml/min. Three polysaccharide fractions (ABP-AW1, ABP-AWA1 and ABP-AWB1) were obtained. The eluted ABP-AW1 was applied to a Sephadex G-25 column (2.6×40 cm; Amersham Biosciences) to remove any salts. Subsequently, ABP-AW1 was collected, dialyzed and lyophilized to obtain the purified polysaccharide for use in the subsequent experiments.

### Immunization protocol

For the immunization, groups of male ICR mice were subcutaneously immunized with 100 μl 0.9% saline containing 100 μg OVA (Sigma, St. Louis, MO, USA) alone, 100 μg OVA + 200 μg alum (Sigma), or 100 μg OVA + ABP-AW1 (50, 100 and 200 μg). Saline-treated mice were included as a control group. Immunizations were performed twice with a two-week interval and the mice were sacrificed two weeks after the secondary immunization. Blood was collected and centrifuged at 3,000 × g for 10 min to generate serum samples, which were stored at −80°C, and splenocyte suspensions were collected under aseptic conditions.

### Splenocyte proliferation assay in vitro

Single-cell suspensions in RPMI-1640 were collected from the OVA-immunized mice under aseptic conditions. Splenocyte proliferation was assayed as previously described (28). Briefly, single-cell suspensions of OVA-immunized mouse spleens were created by grinding the spleens with a syringe plunger against a fine steel mesh. The erythrocytes were lysed with ammonium chloride (0.8%, w/v) and centrifuged at 300 × g at 4°C for 10 min. Mononuclear cells were washed three times in PBS and re-suspended in FCS-RPMI. Splenocytes were plated in triplicate in 96-well plates (Corning Inc., Corning, NY, USA) at a concentration of 2×10^6^ cells/ml at 37°C in a humidified 5% CO_2_ incubator, together with various concentrations of OVA (10 μg/ml), concanavalin A (ConA; 5 μg/ml; Sigma) or lipopolysaccharides (LPS; 2 μg/m; Sigma) for 72 h. Proliferative responses were assessed after 72 h. In the last 4 h of each culture, the cultures were pulsed with 20 μl 4 mg/ml MTT (Sigma) and the absorbance was measured using a microplate reader at 570 nm with a 630 nm reference. The stimulation index (SI) was calculated based on the following formula: SI = absorbance value for mitogen-cultures / absorbance value for non-stimulated cultures. Statistical significance was determined using the Student’s t-test.

### Measurement of OVA-specific IgG and subclasses

OVA-specific IgG and subclass levels in the serum were determined with an indirect enzyme-linked immunosorbent assay (ELISA) according to the previously described method, with slight modifications (29). In brief, round-bottom 96-well high-binding microtiter plates were coated with 100 μl OVA solution (50 μg/ml in 50 mM carbonate-bicarbonate buffer; pH 9.6) overnight at 4°C. Three washes were performed using PBS-0.05% Tween (PBST) solution, followed by blocking with 200 μl 5% FCS/PBS at 37°C for 2 h. Subsequent to three washes with PBST, 100 μl of a series of diluted serum samples or 0.5% FCS/PBS as a control was added to triplicate wells, followed by a 1-h incubation at 37°C. After being washed, the bound antibody was detected using horseradish peroxidase-conjugated goat anti-mouse IgG (Southern Biotech, Birmingham, AL, USA), IgG1 (Southern Biotech) or IgG2b (Southern Biotech) and the unbound antibodies were removed by washing five times with PBST after 1 h at 37°C. 3,3′,5,5′-Tetramethylbenzidine (TMB) liquid substrate was added and incubated for 15 min at 37°C and the reaction was stopped by adding 2 M H_2_SO_4_/well. The optical density was measured using a microplate reader at 450 nm. Statistical significance was determined using Student’s t-test.

### Cytokine determination in cultured splenocyte supernatants

Splenocytes were isolated from the immunized mice as described previously (30). All cell suspensions were aliquoted into 24-well round-bottom plates at 2×10^6^ cells/well. Samples were incubated at 37°C in 5% CO_2_ with OVA (final concentration 10 μg/ml). After 48 h, the plates were centrifuged at 1,400 × g for 5 min and the presence of IFN-γ was measured in the cultured supernatants of the splenocytes using the mouse IFN-γ ELISA kits (R&D Systems, Wiesbaden, Germany). Statistical significance was determined using Student’s t-test.

### Cell surface and intracellular cytokine staining

The cell surface and intracellular cytokine staining was evaluated using flow cytometry (31,32). Briefly, cell suspensions from immunized mice were harvested from the spleen as described previously, then splenocytes were aliquoted into six-well round-bottom plates at 1×10^6^ cells/well and stimulated with 10 μg/ml OVA in the presence of 5 μM brefeldin A (10 μg/ml; Sigma) for 24 h. The splenocytes were subsequently surface stained with 20 μl anti-mouse CD4-FITC Ab (BD Pharmingen, San Diego, CA, USA) and 20 μl CD69-PE/Cy5.5 Ab (BD Pharmingen) diluted in FACS buffer (0.5% bovine serum albumin in phosphate-buffered saline) for 30 min at 4°C. The cells were then washed with FACS, fixed with 1 ml 4% paraformaldehyde and incubated for 10 min at 4°C. The cells were washed three times with 1 ml PBS, then permeabilized with 0.1% saponin for 2–3 min at 4°C. After centrifugation, intracellular staining was performed with anti-mouse IFN-γ-Alexa Fluor 647 (20 μl; BD Pharmingen), followed by incubation for 30 min at 4°C and flow cytometry analysis, which was performed using the FlowJo software (Tree Star, Ashland, OR, USA).

### Statistical analysis

The data are expressed as the mean ± SD. Student’s t-tests were conducted to analyze the differences among the means using SPSS 13.0 software (SPSS, Inc., Chicago, IL, USA). P<0.05 was considered to indicate a statistically significant difference.

## Results

### Effect of ABP-AW1 on the proliferation of splenocytes from OVA-immunized mice

**Figure 1:**
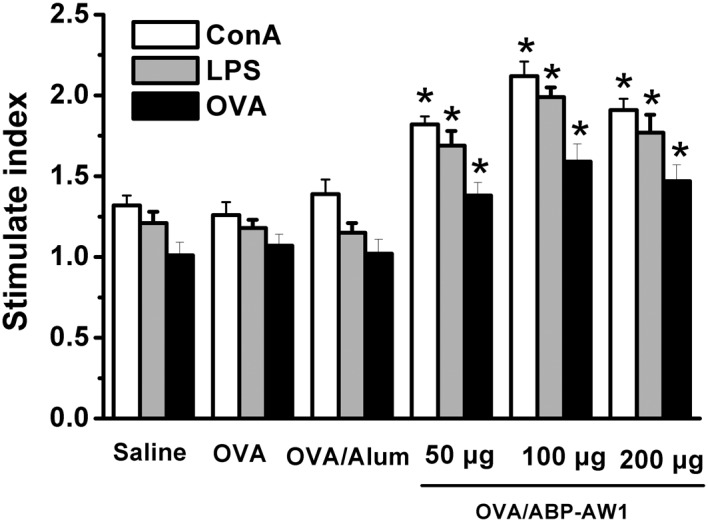
Effect of ABP-AW1 extracted from *Agaricus blazei* on ConA-, LPS- and ovalbumin (OVA)-stimulated proliferation of murine splenic lymphocytes *in vitro.* Data are plotted as the mean ± SD (n=5) of three independent experiments with splenic lymphocytes from different mice. Significant differences from the OVA and OVA/alum groups were designated as ^*^P<0.05. Alum, aluminum; Con A, concanavalin A; LPS, lipopolysaccharides.

To investigate the effect of ABP-AW1 on OVA-immunized mice, the proliferation of these cells was analyzed in the spleens of OVA-immunized mice. It was observed that the mice immunized with OVA/ABP-AW1 had significant immunological responses (Fig. 1). ConA, LPS and OVA stimulated the proliferation of splenic lymphocytes in the mice immunized with OVA/ABP-AW1, and the response was significantly larger than in the OVA or OVA/alum groups (P<0.05), particularly with ABP-AW1 at a dose of 100 μg. However, no significant differences were detected among the saline, OVA alone and OVA/alum groups (P>0.05).

### Effect of ABP-AW1 on OVA-specific serum antibody response

**Figure 2:**
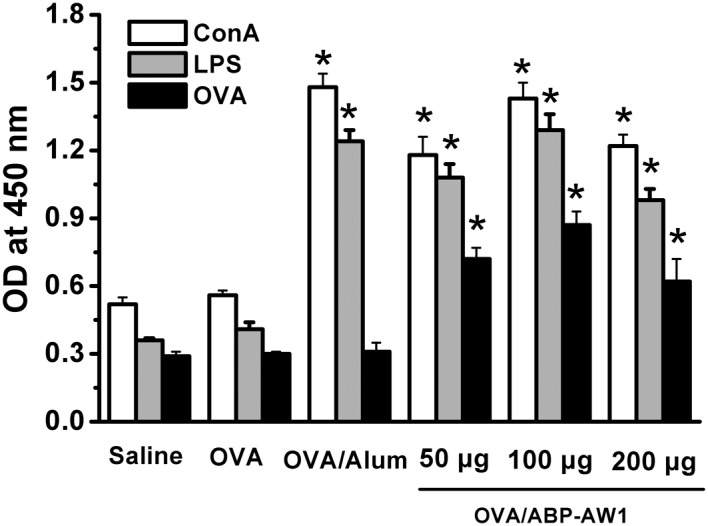
Effect of ABP-AW1 extracted from *Agaricus blazei* on ovalbumin (OVA)-specific IgG, IgG1 and IgG2b antibody levels in OVA-immunized mice. Data are plotted as the mean ± SD (n=5) of triplicate wells. Significant differences with from the OVA and OVA/alum groups were designated as ^*^P<0.05. Alum, aluminum; Con A, concanavalin A; LPS, lipopolysaccharides.

The effects of ABP-AW1 on OVA-specific IgG, IgG1 and IgG2b antibody production were examined using the methods described previously. The total levels of IgG antibody (Fig. 2) were elevated significantly in the OVA/alum and OVA/ABP-AW1 groups compared with the OVA alone group. The IgG1 subtype is reported to be associated with Th2-dominated immune responses, whereas IgG2b is considered to be a mediator of Th1-type immunity (33). Fig. 2 shows that the administration of OVA/ABP-AW1 markedly augmented the production of the OVA-specific IgG1 and IgG2b isotypes. Moreover, considerable enhancements were detected in OVA-specific IgG2b levels in mice immunized with OVA/ABP-AW1 compared with the OVA group (P<0.05), and significant differences were observed among the ABP-AW1 groups at all doses (P<0.05). As a positive control, alum elicited the highest IgG and IgG1 isotype levels, but did not function at the IgG2b level. These data indicate that the Th1 and Th2 immune responses were induced by the OVA/ABP-AW1 vaccine *in vivo* and that the ABP-AW1 adjuvant is required to induce Th1-biased IgG isotype switching.

### Splenic CD4^+^ activation assay

**Figure 3:**
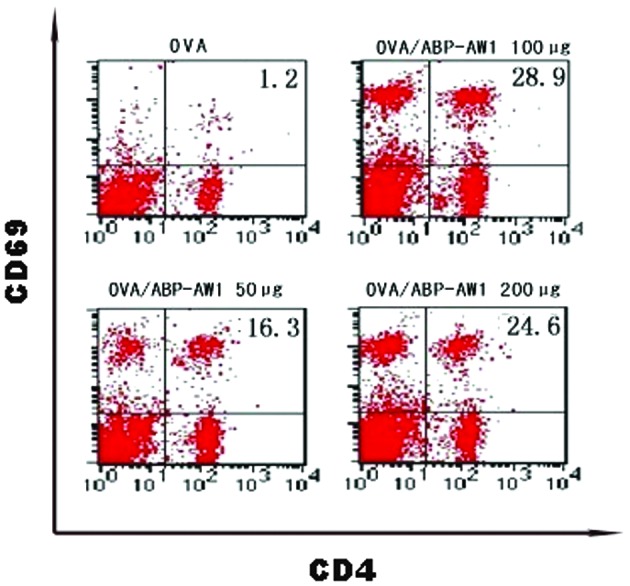
Effect of ABP-AW1 extracted from *Agaricus blazei* on the expression of CD69 in splenocytes extracted from the ovalbumin (OVA)-immunized mice. The activation of CD69^+^ CD4 cells was examined by flow cytometry. The numbers indicate the percentage of cells in the quadrant.

The recall response of T helper cells was assayed two weeks after the final booster vaccination. The phenotype of the CD4^+^ T cells was characterized in the immunized mice in order to determine whether CD4^+^ T cells express activation markers. The early activation marker CD69 was detected on the splenic CD4^+^ T cells. It was observed that the ratio of activated T cells (CD4^+^CD69^+^) to CD4^+^ T cells in the OVA/ABP-AW1 immunized group was higher compared with the OVA alone group (Fig. 3), indicating that OVA/ABP-AW1 induced marked antigen-specific induction of splenic CD4^+^CD69^+^ T cells in comparison with OVA stimulation.

### Effect of ABP-AW1 on cytokine levels in splenocytes from OVA-immunized mice

**Figure 4:**
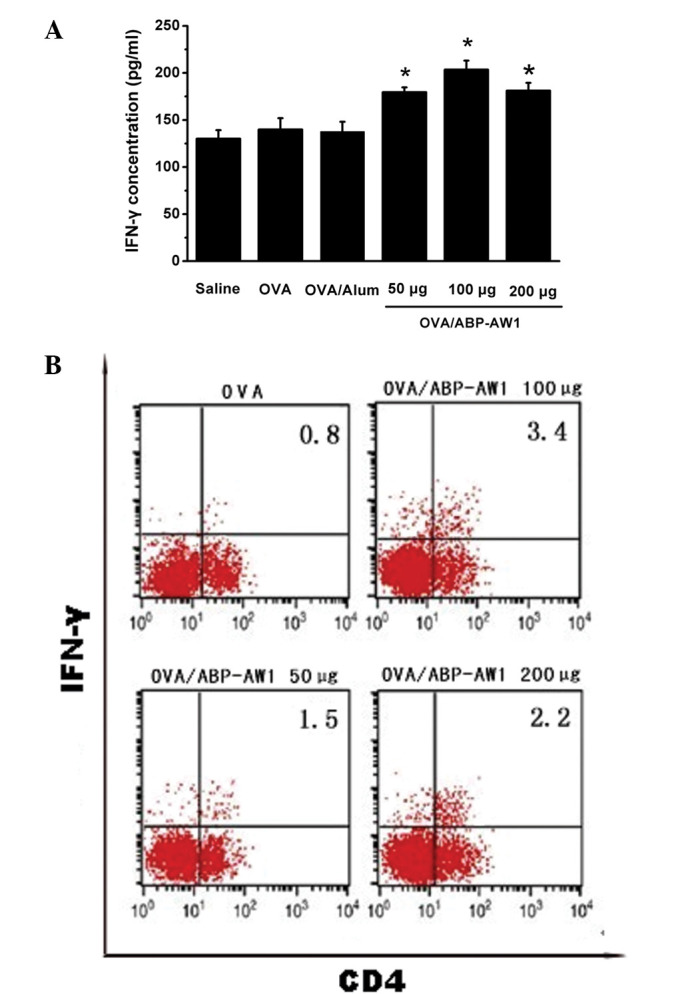
Effect of ABP-AW1 extracted from *Agaricus blazei* on cytokine production in splenocytes from the ovalbumin (OVA)-immunized mice. (A) Levels of IFN-γ in the culture supernatants were tested by ELISA. The values are expressed as the mean ± SD. Significant differences from the OVA and OVA/alum groups were designated as ^*^P<0.05. (B) Production of IFN-γ on OVA-specific CD4^+^ T cells was examined using an intracellular cytokine staining method with flow cytometry. The numbers indicate the percentage of cells in the quadrant. Alum, aluminum.

The previously mentioned findings suggested that ABP-AW1 has immunomodulation activity as an adjuvant. To investigate this further, ELISA analysis was performed to determine the expression patterns of the immune response in the immunized mice. Compared with the levels of cytokines in the culture supernatant of splenocytes from OVA-immunized mice, it was observed that the OVA/ABP-AW1 vaccine had differential cytokine expression profiles. The culture supernatant of splenocytes from the OVA/ABP-AW1-immunized mice showed high levels of IFN-γ (Th1 type immune response; Fig. 4A) in comparison to the OVA/alum or OVA alone groups. Consistent with the ELISA results, the intracellular FACS analysis showed that the CD4^+^ T cell populations from the OVA/ABP-AW1 group expressed significantly higher levels of IFN-γ compared with the OVA control group (Fig. 4B). The proportion of IFN-γ^+^CD4^+^ (double-positive) T cells from the mice immunized with OVA/ABP-AW1 was markedly increased. The CD4^+^ T cells obtained from the mice immunized with OVA/alum showed negligible IFN-γ production (data not shown). Thus, it may be suggested that OVA/ABP-AW1 markedly induces Th1 cells, as determined by IFN-γ production. These results showed noticeable correlations, indicating that the addition of ABP-AW1 to OVA enhances Th1 polarization.

## Discussion

The present study demonstrated the *in vivo* efficacy of ABP-AW1 as an adjuvant for OVA-based vaccines. Ideally, adjuvants should promote an appropriate immune response, (Th1 or Th2), be biodegradable and not be immunogenic themselves. Adjuvants should also offer excellent safety, tolerability, ease of manufacture and formulation. Therefore, modern adjuvants should have a profound effect on the nature of the immune response and it is often desirable to induce specific types of immunity (34), which may bias the immune system toward either a Th1 or a Th2 type response (35). Among the T lymphocytes, T cell responses have been divided in two subclasses, Th1 and Th2. Th1 and Th2 immune response profiles correspond to the activation of two distinct major subsets of T cells characterized by their pattern of cytokine production (29,33), i.e. IL-2, TNF-β and IFN-γ vs. IL-4, IL-5 and IL-10, respectively. Th1 cells protect against intracellular pathogens, activate phagocytosis, induce IgG2a, IgG2b and IgG3 antibodies and promote delayed-type hypersensitivity responses, whereas Th2 cells protect against extracellular pathogens, activate eosinophils, induce IgE-mediated allergic responses and promote other humoral responses in which IgG1, IgGE and IgA predominate (35). Polarized Th1 and Th2 phenotypes are critical in the course of immune responses, and efficient Th1 and Th2 immunity-inducing adjuvants, particularly those for Th1, are highly in demand (2). Such adjuvants promote good cell-mediated immunity. The development of such adjuvants would benefit from increased knowledge of the molecular mechanisms and factors controlling these responses.

The present study evaluated whether ABP-AW1 was able to enhance immunity to OVA in mice, providing insight into the OVA/ABP-AW1 adjuvant system and *in vivo* evidence. The results showed that ABP-AW1 was sufficient to enhance the activation potential of T and B lymphocytes in OVA-immunized mice. Furthermore, antigen-specific CD4^+^ helper T cells are also regarded as an important attribute of ABP-AW1 responses, which are directly associated with immunostimulation. To the best of our knowledge, CD69 is a C-type lectin that is expressed on the surface of all leukocytes during activation, and the engagement of CD69 maintains the high expression of membrane-bound TGF-β1 on T cells (36). The early activation marker CD69 was detected on CD4^+^ T cells, suggesting that their generation requires T cell activation. Adaptive major histocompatibility complex (MHC)-II-mediated CD4^+^ T cell activation has been considered to be strictly restricted to protein antigens (37). When presented to T cells by MHC-II, antigens generally trigger a T cell-dependent immune response typified by the production of Th1 or Th2 cytokines, as well as IgG and the induction of immunological memory. However, the T-lymphocyte-independent nature of a polysaccharide antigen may be overcome by conjugating polysaccharides to protein carriers (38,39). Such conjugates have been demonstrated to be efficient in inducing T-lymphocyte-dependent immunity, as well as protecting infants and the elderly from infection (40). The present study demonstrated that OVA/ABP-AW1 activated T cells. This result was consistent with the increased numbers of splenic CD4^+^CD69^+^ T cells observed in the OVA-immunized mice (Fig. 3). ABP-AW1 induced marked antigen-specific activation of the splenic CD4^+^CD69^+^ T cells in response to OVA stimulation, indicating that the OVA/ABP-AW1 vaccine was efficient in inducing T-lymphocyte-dependent immunity.

Evidence is accumulating that ABP-AW1 is a potential new adjuvant. Cytokines have a central role during Th1 immune adjustment and IFN-γ is a critical cytokine that coordinates the immune response through the transcriptional regulation of immunologically relevant genes, inducing a wide variety of antigen-presenting cells to express MHC-I and MHC-II molecules, promoting the efficacy of antigen-specific T cell activation and macrophage phagocytosis and stimulating B cells to produce antibodies (41). IFN-γ is involved in a wide range of infectious diseases and cancer immunotherapy. As shown in Fig. 4, ABP-AW1 elicited a marked Th1-polarized immune response to OVA in the immunized mice. The level of the cytokine, IFN-γ, in the cultured supernatants from the splenocytes was significantly enhanced by OVA/ABP-AW1 compared with OVA alone. Th1 polarization was also characterized by CD4^+^ T-cell cytokine release profiles, as demonstrated by the high levels of IFN-γ produced in response to antigen restimulation. The humoral immune response induced by the OVA/ABP-AW1 vaccine was also determined in accordance with Th1 polarization. The key finding of the present study was that the OVA/ABP-AW1 vaccine was able to induce high levels of IgG (total), IgG1 and IgG2b antibodies (Fig. 2), with the addition of ABP-AW1 to OVA at a suitable dose being effective on Th1 and Th2 cells, resulting in a mixed Th1/Th2 immune response. However, as a positive control group OVA/alum generated lower levels of IgG2b compared with OVA/ABP-AW1. ABP-AW1 had the benefit over alum in that it also stimulated cellular immunity as reflected by Th1 antibody IgG2b isotype induction. Collectively, this cascade of immune events provided evidence that ABP-AW1 adjuvant has a key role in inducing a marked Th1 immune response to OVA antigen.

The efficacy of OVA antigen was greatly enhanced by formulation with ABP-AW1. This may be explained by investigating the various types of pathways by which ABP-AW1 may stimulate the immune system. For example, the ABP-AW1 response may be derived from NK cells stimulating IFN-γ production at the injection site (13,42), which may result in the increased activation of dendritic cells (DCs) that are taking up antigen. In turn, DCs stimulate IFN-γ-producing cells, potentially prolonging the release from the NK cells and T cells, while IFN-γ further substantially enhances IgG2b and IgG2a production by effecting the isotype switching of the B-lymphocytes (33,35). Another possible pathway is the spread of ABP-AW1 into the circulation, whereby ABP-AW1 likely acts systemically on the NK or T cells to stimulate IFN-γ production or the clonal expansion of the T cells. In addition, a low incidence of adverse events is critical for the advancement of vaccine adjuvant candidates; ABP-AW1 is extracted from *Agaricus blazei* Murill, which is edible to humans without ill effects (25). While further studies are required to clarify the relative contributions of these pathways, the present data showed that ABP-AW1 could be selected and used as an adjuvant for the modulation of immune responses.

In conclusion, the present study evaluated the adjuvant activity of ABP-AW1 extracted from *Agaricus blazei* Murill. The present data indicate that ABP-AW1 is a candidate immunity-stimulating adjuvant and that ABP-AW1 particularly promotes the development of Th1 polarization. Future studies should aim to evaluate this adjuvant for use in vaccines instead of alum. Moreover, the mechanism of the effects of ABP-AW1 as an adjuvant must be elucidated more specifically.
